# A dataset for automatic contrast enhancement of microscopic malaria infected blood RGB images

**DOI:** 10.1016/j.dib.2019.104643

**Published:** 2019-10-12

**Authors:** J. Somasekar, G. Ramesh, Gandikota Ramu, P. Dileep Kumar Reddy, B. Eswara Reddy, Ching-Hao Lai

**Affiliations:** a Department of Computer Science and Engineering, Gopalan College of Engineering and Management, Whitefield, Bangalore, Karnataka, 560 048, India; b Department of CSE, GRIET, Bachupally, Hyderabad, Telangana, 500 090, India; c Department of CSE, Institute of Aeronautical Engineering, Hyderabad, Telangana, 500043, India; d Department of CSE, Sri Venkateswara College of Engineering, Karakambadi Road, Tirupati, 517 507, India; e Department of CSE, JNTUA College of Engineering Ananthapuramu, Anantapuramu, Andhra Pradesh, 515 002, India; f Computational Intelligence Technology Center, Industrial Technology Research Institute, Chung Hsing Rd., Chutung, Hsinchu, 310 40, Taiwan

**Keywords:** Malaria diagnosis, Low contrast images, Histogram equalization, Microscopic RGB blood images, Contrast enhancement

## Abstract

In this article we introduce a malaria infected microscopic images dataset for contrast enhancement which assist for malaria diagnosis more accurately. The dataset contains around two hundred malaria infected, normal, species and various stages of microscopic blood images. We propose and experimentally demonstrate a contrast enhancement technique for this dataset. This simple technique increases the contrast of an image and hence, reveals significant information about malaria infected cells. Experiments on the dataset show the superior performance of our proposed method for contrast enhancement of malaria microscopic imaging.

**Table:** Specifications Table

Subject area	Computer science, medical imaging
More specific subject area	Medical imaging
Type of data	Images, Graphs
How data was acquired	Original RGB microscopic blood images are taken from existing public databases (CDC) and Image acquisition Toolbox in MATLAB
Data format	RGB and JPG
Experimental factors	Low contrast and Histogram of an image
Experimental features	Exposure, mean, contrast, enhancement of each component of RGB image, maxima and minima.
Data source location	Data is available in public repository: link https://www.cdc.gov/dpdx/malaria/index.html#tabs-1-2
Data accessibility	The data are available with this article and accessible to the public

**Table:** 

**Value of the Data** • Data could be used as an initial data for further experiment in automatic malaria diagnosis. • The dataset can be used to test machine learning classification methods. • The data can be used in microscopic image analysis. • This data set may encourage further research on computer aided diagnosis (CAD) system. • The data set is very useful to train classification system for species of malaria parasites.

## 1 Data

**Fig. 1:**
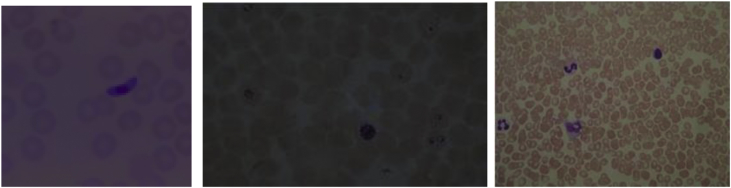
Low contrast malaria infected microscopic blood images.

**Fig. 2:**
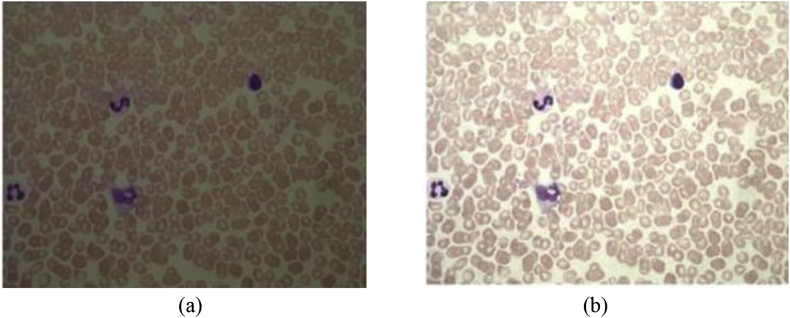
(a) Original low contrast image from dataset, (b) Contrast enhanced image by proposed method.

**Fig. 3:**
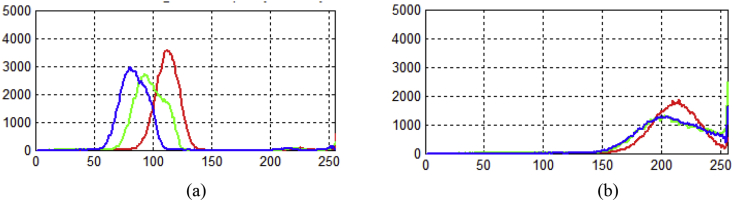
(a) Histogram of original image as shown in Fig. 2(a), (b) histogram of contrast enhanced image as shown in Fig. 2 (b).

**Fig. 4:**
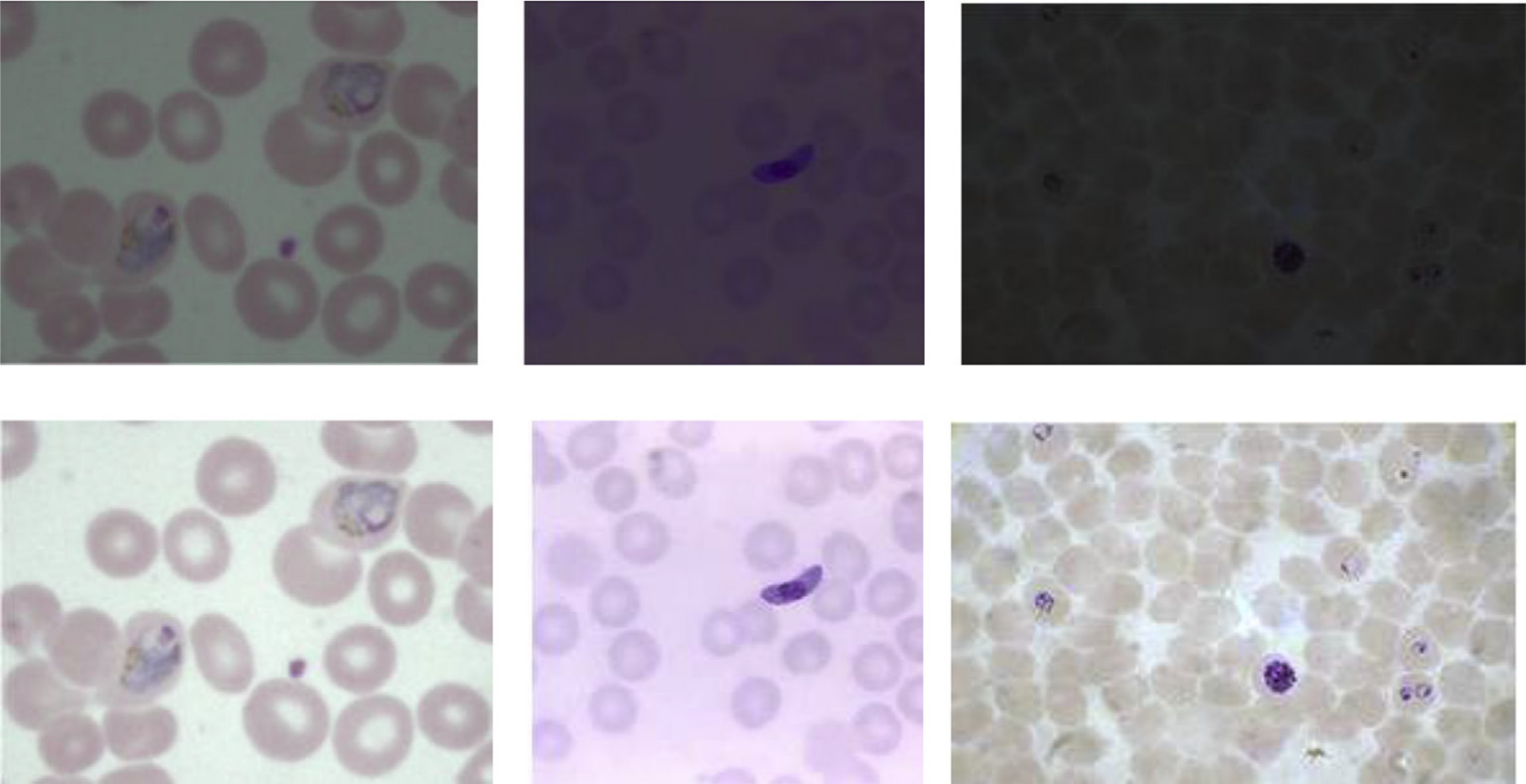
First row indicates low contrast malaria microscopic images at various stages (Ring, Gametocyte, Trophozoite) from dataset and second row indicates the contrast enhancement of the first row images by proposed method.

**Fig. 5:**
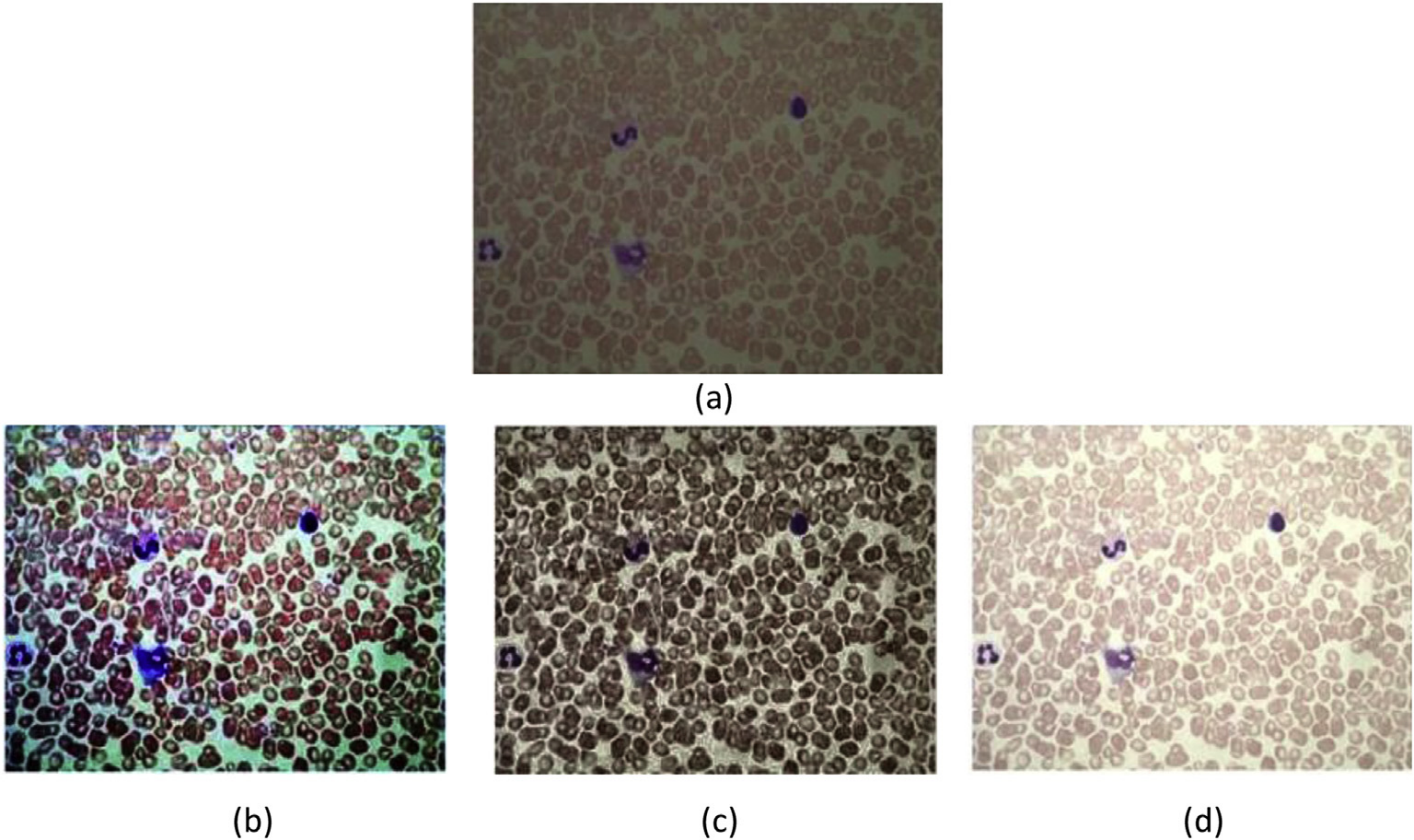
Enhanced results of a low contrast color microscopic malaria image using different methods. (a) Original image (b) HE (c) CLAHE and (d) proposed.

The dataset in this article describes the malaria infected cells microscopic imaging with various contrast environments. It is difficult to identify the infected cells through automation and manually when the image having low contrast which leads to false diagnosis [2,4]. Fig. 1 describes the low contrast malaria infected microscopic images. Fig. 2 describes the low contrast malaria infected microscopic image and enhanced image by proposed method. Fig. 3 describes the histograms of original and enhanced RGB images. Fig. 4 describes contrast enhancement of low contrast malaria infected images at various stages. Fig. 5 displays comparison of proposed method with other existing methods on contrast enhancement of malaria microscopic imaging.

## 2 Experimental design, materials and methods

This section brings a brief explanation about the data set used, experiment processing and methodology of the proposed method for contrast enhancement of microscopic blood imaging.

### 2.1 Data set and experiment processing

The microscopic malaria images dataset for automatic contrast enhancement was performed using MATLAB 7.10.0 (R2010a) Programming software on a personal computer with an AMD Phenom II N830 triple-core processor 2.10 GHz, 3 GB system memory and 64-bit windows-7 operating system. The dataset from the Centers for Disease Control and Prevention (CDC) [https://www.cdc.gov/dpdx/malaria/index.html#tabs-1-2] have been used for the experiments [1]. Few more malaria images in dataset were supplied by Dr. Ashok K. Maiti, Department of Pathology, Midnapur Medical College & Hospital, Midnapur, West Bengal, India and few tested images from research articles [2,8,11].

### 2.2 Methods

Microscopic blood images are commonly acquired using a digital camera with a blood smear attachment. The input blood color image f(x,y) of size m×n has three channels Red, Green and Blue, is denoted by the following mathematical expression.

(1) $$\;f\left(x,y\right)=\left(\begin{array}{c} f_{R}\left(x,y\right)\\ f_{G}\left(x,y\right)\\ f_{B}\left(x,y\right) \end{array}\right)$$

where, (x,y)∈{0,1,2,…,m−1}×{0,1,2,…,n−1}

To transfer the imagef(x,y) into an image g(x,y) so that it retains all the relevant information of the original image to improve the contrast of the image. Therefore, the image g(x,y) is contrast enhanced image which is considered as the standard version of the original image for further processing which assisting to improve the performance of the diagnosis. The three channels in the contrast enhanced image g(x,y) of an imagef(x,y), are constructed by using the following proposed mathematical equation [2,3].

(2) $$g\left(x,y\right)=\left(\begin{array}{c} g_{R}\left(x,y\right)\\ g_{G}\left(x,y\right)\\ g_{B}\left(x,y\right) \end{array}\right)$$

$$\;g\left(x,y\right)=D{\left(\left[\begin{array}{c} \left[\frac{f_{R}^{max}\left(x,y\right)-f_{R}^{min}\left(x,y\right)}{f_{R}^{max}\left(x,y\right)+f_{R}^{min}\left(x,y\right)}\right]\\ \left[\frac{f_{G}^{max}\left(x,y\right)-f_{G}^{min}\left(x,y\right)}{f_{G}^{max}\left(x,y\right)+f_{G}^{min}\left(x,y\right)}\right]\\ \left[\frac{f_{B}^{max}\left(x,y\right)-f_{B}^{min}\left(x,y\right)}{f_{B}^{max}\left(x,y\right)+f_{B}^{min}\left(x,y\right)}\right] \end{array}\right]+\left[\begin{array}{c} \delta_{R}\\ \delta_{G}\\ \delta_{B} \end{array}\right]\right)}^{T}⊗\left(D{\left(\begin{array}{c} f_{R}\left(x,y\right)\\ f_{G}\left(x,y\right)\\ f_{B}\left(x,y\right) \end{array}\right)}^{T}⊗\left(\begin{array}{c} {\left[{\left(mn\right)}^{-1\;}\left(\overset{m-1}{\underset{x=0}{\sum }}\overset{n-1}{\underset{y=0}{\sum }}f_{R}\left(x,y\right)\right)\right]}^{-1}\\ {\left[{\left(mn\right)}^{-1\;}\left(\overset{m-1}{\underset{x=0}{\sum }}\overset{n-1}{\underset{y=0}{\sum }}f_{G}\left(x,y\right)\right)\right]}^{-1}\\ {\left[{\left(mn\right)}^{-1\;}\left(\overset{m-1}{\underset{x=0}{\sum }}\overset{n-1}{\underset{y=0}{\sum }}f_{B}\left(x,y\right)\right)\right]}^{-1} \end{array}\right)\right)$$

whereD(.) is a diagonal matrix, the operator ⊗ is a matrix multiplication and T indicates transpose of a matrix. In the above equation, the parameter $\delta_{\vartheta }$ is to control the level of contrast and is obtained by using the equation (3). Where, ϑ∈{R,G,B}

(3) $$\;\left(\begin{array}{c} \delta_{R}\\ \delta_{G}\\ \delta_{B} \end{array}\right)=\left(\begin{array}{c} \frac{9f_{R}^{min}\left(x,y\right)-f_{R}^{max}\left(x,y\right)}{5f_{R}^{max}\left(x,y\right)+5f_{R}^{min}\left(x,y\right)}\;\\ \;\frac{7f_{G}^{min}\left(x,y\right)-f_{G}^{max}\left(x,y\right)}{4f_{G}^{max}\left(x,y\right)+4f_{G}^{min}\left(x,y\right)}\\ \frac{9f_{B}^{min}\left(x,y\right)-f_{B}^{max}\left(x,y\right)}{5f_{B}^{max}\left(x,y\right)+5f_{B}^{min}\left(x,y\right)} \end{array}\right)$$

The values for $f_{\vartheta }^{max}\left(x,y\right)$and $f_{\vartheta }^{min}\left(x,y\right)$ are defined by.

$f_{\vartheta }^{max}\left(x,y\right)=\text{max}\left\{\;f_{\vartheta }\left(x,y\right):0\leq x\leq m-1,0\leq y\leq n-1\right\}$
$f_{\vartheta }^{min}\left(x,y\right)=\min\left\{f_{\vartheta }\left(x,y\right)\;:0\leq x\leq m-1,0\leq y\leq n-1\right\}$

In Assessment of visual quality, Fig. 2 (a) shows the low contrast image from dataset and Fig. 2 (b) shows the contrast enhanced image. By observing the resultant image, we can find that the proposed method yields a better and good contrast image which assists diagnosis. Beside, the histograms for the original image and enhanced images as shown in Fig. 3. The contrast enhancement of various stages ie., Ring, Gametocyte, Trophozoite of malaria low contrast microscopic images from dataset are shown in Fig. 4. The median filter is used to reduce the noise before enhancement [5]. In order to evaluate the performance of the proposed method with other existing methods quantitatively by edge-based contrast measure (EBCM) [[10], [11], [12], [13], [14], [15]]. The EBCM for original image f(x,y) as shown in Eqn. (4).

(4) $$EBCM\left[f\left(x,y\right)\right]=\overset{m}{\underset{x=1}{\sum }}\overset{n}{\underset{y=1}{\sum }}C\left(x,y\right)\;\;/\;\overset{L-1}{\underset{\;k=0}{\sum }}H\left(k\right)\;=\frac{1}{mn}\;\overset{m}{\underset{x=1}{\sum }}\overset{n}{\underset{y=1}{\sum }}C\left(x,y\right)$$

Analogous definition can be given for enhanced imageg(x,y).

In this study, the proposed technique is be compared with some other existing contrast enhancement methods, which includes Histogram Equalization (HE) and Contrast Limited Adaptive Histogram Equalization (CLAHE) [[6], [7], [8], [9]]. Table 1 shows EBCM for the tested images using various methods. Table 1 reveals that the EBCM value of the proposed method have higher values than original image and ensure for good and natural enhancement of image when compared to other methods. The contrast enhancement results of the proposed with existing standard methods HE, CLAHE resultant images are shown in Fig. 5. The proposed yields better contrast enhancement when compared to the existing methods. The proposed method works well when an image suffers from artifacts and noise.

**Table 1:** Quantitative measurement results as EBCM.

Image ID	Original	HE	CLAHE	Proposed
1	246.06	179.82	224.41	249.71
2	141.05	133.78	125.22	252.43
3	152.34	144.17	156.29	252.85
4	230.61	232.26	167.43	244.78
5	243.28	213.02	243.211	244.67

Note: More than original image value indicates better enhancement performance.
